# Characterization of binding affinity changes of SARS-CoV-2 omicron variant peptides to population-specific HLA

**DOI:** 10.1186/s12929-025-01139-5

**Published:** 2025-04-29

**Authors:** Che-Mai Chang, Chang-Jiun Wu, Maxim Shkurnikov, Chin-Lin Guo, Wan-Chen Huang, Alexander Tonevitsky, Wei-Chiao Chang

**Affiliations:** 1 Master Program in Clinical Genomics and Proteomics, College of Pharmacy, Taipei Medical University, Taipei, Taiwan; 2 Department of Pharmaceutical Sciences, School of Pharmacy, Taipei Medical University, Taipei, Taiwan; 3 Faculty of Biology and Biotechnology, HSE University, Moscow, Russia; 4 Institute of Physics, Academia Sinica, Taipei, Taiwan; 5 Single-Molecule Biology Core Lab, Institute of Cellular and Organismic Biology, Academia Sinica, Taipei, Taiwan; 6 Art Photonics GmbH, Berlin, Germany; 7 Department of Clinical Pharmacy, School of Pharmacy, Taipei Medical University, No. 250, Wu-Xing St, Taipei, 11031 Taiwan; 8 Department of Medical Education and Research, Integrative Research Center for Critical Care, Taipei Medical University, Wan-Fang Hospital, Taipei, Taiwan; 9 Department of Pharmacy, Wan Fang Hospital, Taipei Medical University, Taipei, Taiwan

**Keywords:** SARS-CoV-2, Omicron variant, Spike protein, T-CoV, HLA

## Abstract

**Background:**

The evolution of SARS-CoV-2, particularly through new variants, presents significant global health challenges due to their potential for immune evasion and reduced vaccine effectiveness. This study aims to investigate the impact of mutations in the Spike protein of Omicron EG.5 and XBB.1.16 variants on the binding affinities of viral peptides to common human leukocyte antigen (HLA) class I and II alleles across Taiwanese, British, and Russian populations. Understanding these interactions is crucial for elucidating differences in immune responses and disease severity among diverse populations.

**Methods:**

We updated the T-CoV portal to incorporate and analyze EG.5 and XBB.1.16 variants. Binding affinities between mutated Spike protein peptides and HLA class I and II alleles were predicted and compared across the three populations. Statistical analyses, including chi-squared tests, were conducted to assess the significance of binding affinity differences across the three populations and between HLA classes.

**Results:**

Our findings revealed that mutations in the Spike protein had a more pronounced effect on HLA class II binding affinities than on HLA class I. While binding affinity profiles for HLA class I were largely consistent across populations, significant population-specific variations were observed for HLA class II alleles. Specifically, the British population exhibited lower proportions of tightly binding mutated peptides compared to the Taiwanese and Russian populations. Furthermore, substantial differences were identified in the binding affinity changes of mutated Spike peptides for HLA class II across Taiwanese, British, and Russian populations, as well as between the Omicron EG.5 and XBB.1.16 variants. Subsequent analyses revealed significant differences in the conservation and evolutionary trajectories of binding affinities between mutated Spike peptides and common HLA class II alleles, both between the EG.5 and XBB.1.16 variants and across the three populations for the XBB.1.16 variant.

**Conclusions:**

In summary, Spike protein mutations in SARS-CoV-2 variants significantly influence immune responses by altering HLA-peptide interactions, with pronounced population-specific effects on HLA class II alleles. These findings underscore the critical role of HLA class II diversity in shaping immune responses and susceptibility to COVID-19. Integrating population-specific HLA profiles into vaccine development and public health strategies is essential for improving interventions against evolving SARS-CoV-2 variants.

## Background

Since its emergence in late 2019, the COVID-19 pandemic, caused by SARS-CoV-2 virus, has undergone continuous evolution, with new variants presenting ongoing global health challenges. Despite widespread vaccination efforts, the virus’s high mutation rate has allowed certain variants to evade immune defenses, leading to recurrent waves of infection and diverse disease outcomes worldwide [1].

The identification of genetic predisposition biomarkers for severe COVID-19 remains a critical area of research. A fundamental component of immune defense is the presentation of viral peptides by human leukocyte antigen (HLA) molecules to T cells. HLA genes are highly polymorphic, leading to variations in antigen presentation and immune responses among individuals [2]. This genetic diversity is associated with different susceptibilities to infections and varied disease outcomes [3]. Certain HLA alleles have been correlated with increased susceptibility or severity of COVID-19, often within a population-specific context, underscoring the influence of genetic factors on disease severity [4–8].

Shortly after the onset of the pandemic, novel SARS-CoV-2 variants emerged that significantly altered the clinical trajectory of the disease. These variants introduced new mutations, especially in the Spike protein—a critical target for immune recognition. These mutations can modify viral peptide sequences, potentially affecting their binding affinities to HLA molecules and, in turn, impacting the efficacy of antigen presentation [9]. Research has shown that certain Spike mutations increase viral infectivity and enable immune evasion by altering the interactions between HLA-presented peptides and T cells [10, 11].

In response to these challenges, we introduced T-CoV in 2021, a web portal summarizing how mutations in prevalent SARS-CoV-2 variants impact the binding affinities between viral peptides and major HLA alleles [5]. While many mutations significantly affect these affinities—often in a population-specific manner [5, 12]—the majority of these changes in Delta and Omicron variants are mitigated at the whole-virus level due to the large number of tight-binding SARS-CoV-2 peptides and the presence of multiple HLA genes in individuals [13]. However, a notable exception is the HLA-DRB1*03:01 allele, which lost all tight-binding peptides in the Spike protein due to Omicron BA.1 mutations [13].

Understanding how these mutations affect HLA-peptide interactions is critical, especially given the population-specific distributions of HLA alleles. This knowledge is essential to explaining observed differences in immune responses and disease severities across various ethnic groups and regions [14, 15].

This study aims to investigate how mutations in the Spike protein of EG.5 and XBB.1.16 SARS-CoV-2 variants affect the binding affinities of viral peptides to common HLA class I and class II alleles in Taiwanese, British, and Russian populations. Through analyzing binding affinity profiles and mutation-induced changes, we seek to identify population-specific differences that may influence immune responses and clinical outcomes.

## Materials and methods

### Data collection and analysis

The data of binding affinities between HLA molecules and viral peptides of SARS-CoV-2 variants were obtained from the T-cell COVID-19 Atlas (T-CoV) portal [5]. For this study, we updated the T-CoV portal by adding EG.5 (genome accession id: EPI_ISL_18111433, hCoV-19/USA/TX-TAMGHRC-SHS_97566/2023) and XBB.1.16 (genome accession id: EPI_ISL_17957802, hCoV-19/India/UP-ILSGS21282/2023) variants. Spike peptides of SARS-CoV-2 Omicron EG.5 and XBB.1.16 variants with altered amino acid sequences that differ from the reference Spike protein sequence in the Wuhan strain were defined as mutated Spike peptides. Only mutated Spike peptides that were predicted to bind to common HLA class I and II alleles were included in this study. The prediction and classification of binding affinities between SARS-CoV-2 Spike peptides and HLA class I and II alleles were conducted using the same methodologies described in our previous study, which established the T-CoV portal [5]. Specifically, viral peptide-HLA binding affinities were predicted using netMHCpan-4.1 and netMHCIIpan-4.0 [16] and subsequently categorized into three binding strength groups: tight binding (IC50 affinity ≤ 50 nM), moderate binding (50 nM < IC50 affinity ≤ 500 nM), and weak/no binding (IC50 affinity > 500 nM). Changes in binding affinities were assessed by comparing the classification of mutated Spike peptides in Omicron variants to that of reference Spike peptides from the Wuhan strain. Based on these comparisons, Spike peptide-HLA binding affinity alterations were classified into three categories: increased binding (from weak/no binding to moderate binding, from weak/no binding to tight binding, and from moderate binding to tight binding), decreased binding (from tight binding to moderate binding, from tight binding to weak/no binding, and from moderate binding to weak/no binding), and unchanged binding (from tight binding to tight binding, from moderate binding to moderate binding, and from weak/no binding to weak/no binding). The profile of changes in the binding affinity of sequentially mutated peptides to HLA in the SARS-CoV-2 Omicron variant was constructed based on the starting positions of the mutated peptides aligned with the reference Spike protein sequence of the Wuhan strain.

### Common HLA selection

The common HLA class I and II alleles for different populations was determined based on our previous study [12]. For the common HLA class I, we included HLA-A, HLA-B, and HLA-C alleles in the Taiwanese, British, and Russian populations. For the common HLA class II, both HLA-DRB1 and HLA-DPA1/DPB1 alleles in the Taiwanese and British populations were included, whereas only HLA-DRB1 alleles in the Russian population were analyzed in this study.

### Statistics

The analysis and visualization for this study were conducted using R software (version 4.1.2). Differences in the distribution of peptide-common HLA binding affinity changes across population, between SARS-CoV-2 Omicron variants, and between HLA classes were analyzed using the chi-squared test. *P*-values less than 0.05 are considered indicative of statistical significance.

## Results

### Comparison of the binding affinity profiles of mutated Spike peptides to common HLA alleles between SARS-CoV-2 Omicron variants and across different populations

**Fig. 1:**
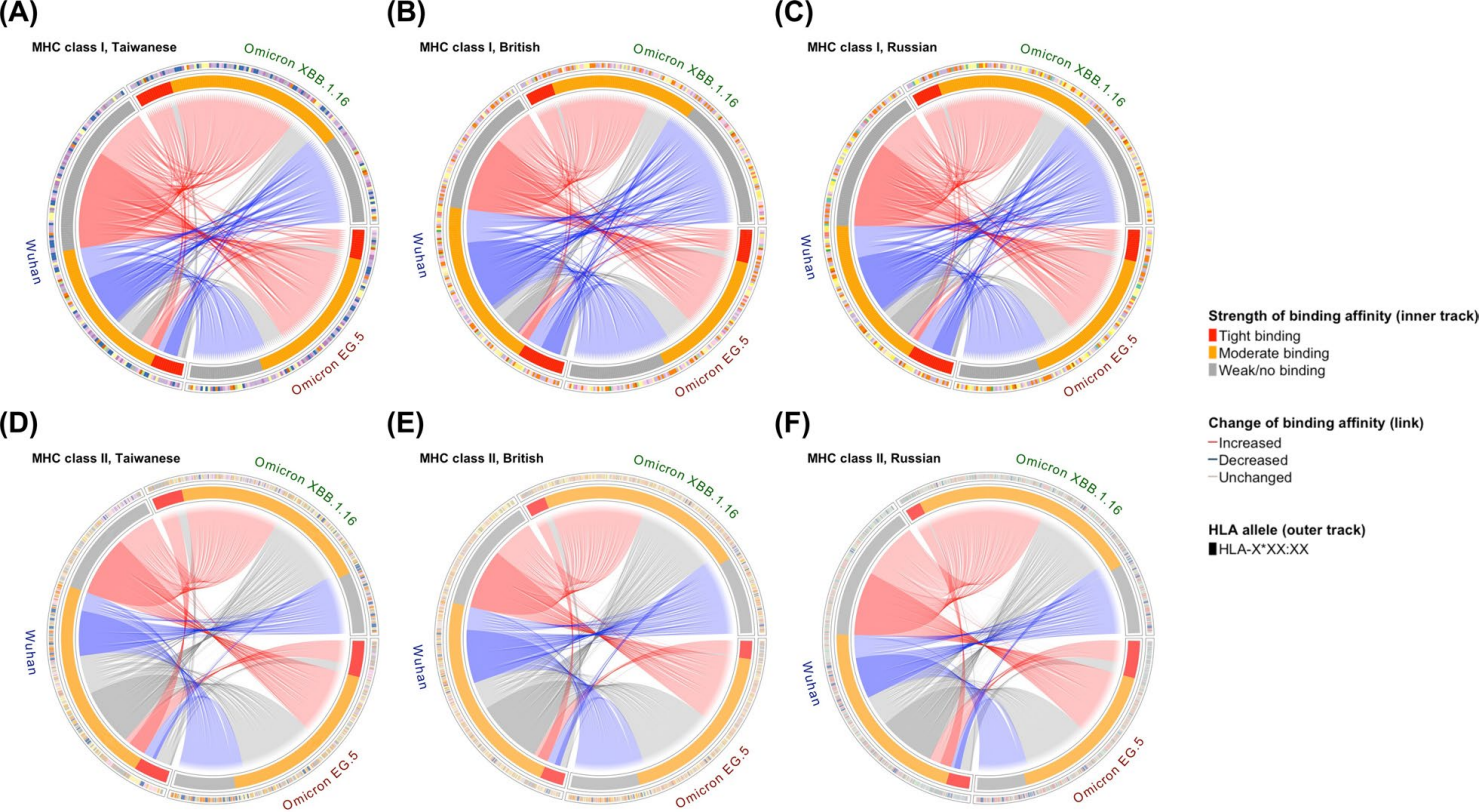
Profiles of mutated Spike peptide-common HLA binding affinity changes of SARS-CoV-2 Omicron variant in different populations. The strengths and changes in binding affinities of Spike peptides to population-specific common HLA class I (**A**–**C**) and II (**D**–**F**) alleles in the Taiwanese (**A**, **D**), British (**B**, **E**), and Russian (**C**, **F**) populations are shown as circus plots. The changes in peptides-HLA binding affinities between the original Wuhan strain and Omicron variants, EG.5 and XBB.1.16, were categorized as increased (red), decreased (blue), and unchanged (gray) alteration and indicated by the links of the plots. The strengths of peptide-HLA binding affinities before and after mutations in the Wuhan strain and Omicron EG.5 or XBB.1.16 variants, respectively, were classified into strong (red), medium (orange), and weak/no (gray) binding, as depicted in the inner track of the plots. The types of HLA alleles were illustrated by different colors, as shown in the outer track of the plots

In the present study, we analyzed and compared the binding affinities of mutated Spike peptides in SARS-CoV-2 variants to common HLA molecules across Taiwanese, British, and Russian populations. Comparing the Omicron variants EG.5 and XBB.1.16 to the original Wuhan strain, the binding affinity profiles for both HLA class I and II were similar across the three populations, but variations were still observed (Fig. 1).

**Fig. 2:**
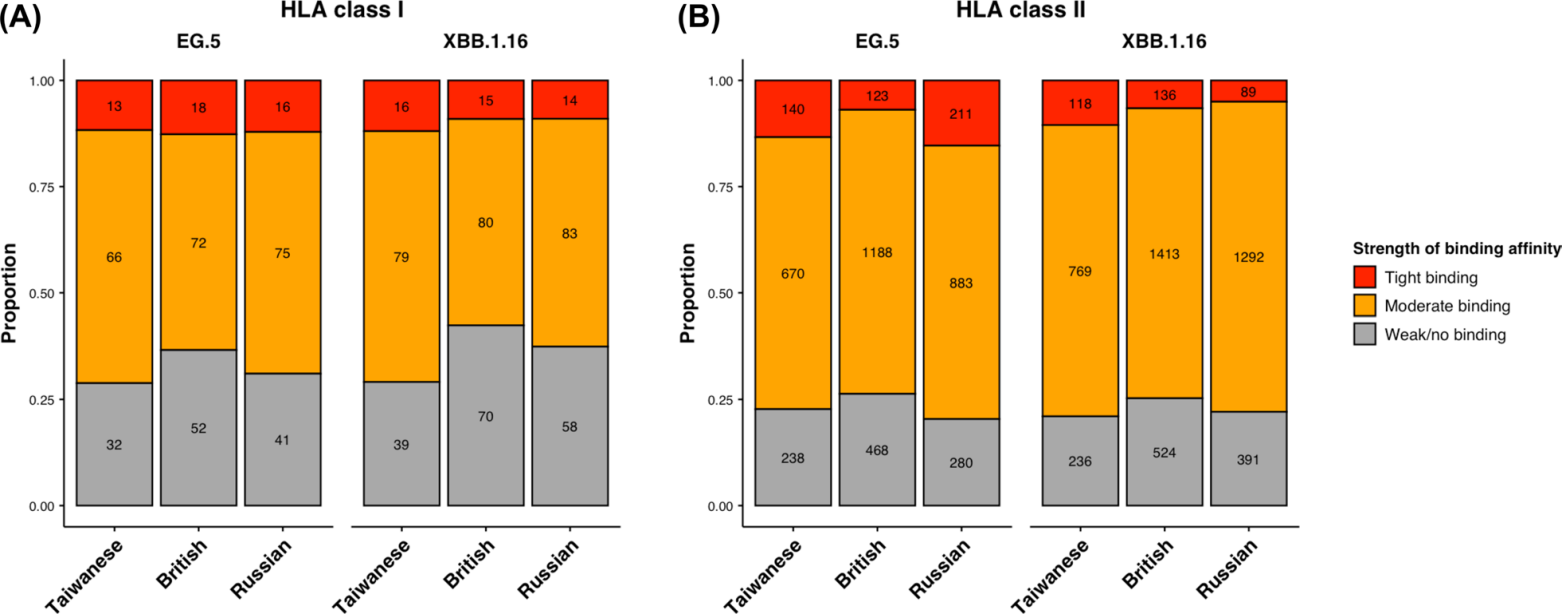
Distributions of mutated Spike peptide-common HLA binding affinity strengths of SARS-CoV-2 Omicron variants across different populations. The binding affinities of mutated Spike peptides from the Omicron EG.5 and XBB.1.16 variants to population-specific common HLA class I (**A**) and II (**B**) alleles in the Taiwanese, British, and Russian populations were determined to be either tight binding (red), moderate binding (orange), or weak/no binding (gray). The proportions of mutated Spike peptide-common HLA binding affinity strengths of individual Omicron variants in different populations were illustrated in separate stacked bars

Interestingly, the binding affinity changes for EG.5 and XBB.1.16 differed between HLA class I and II. To explore this further, we compared the distribution of mutated Spike peptide-common HLA binding affinities across different populations and between two Omicron variants. For HLA class I, there were no significant differences in the proportion of mutated Spike peptides with tight binding affinities to common HLA alleles from EG.5 and XBB.1.16 across the three populations (Fig. 2A). Although the British population showed lower proportions of moderate-binding affinities and higher proportions of weak/no-binding affinities to HLA class I alleles in mutated Spike peptides from both EG.5 and XBB.1.16, statistical analysis revealed no significant difference in the distribution of mutated Spike peptide-common HLA class I binding affinity strengths across Taiwanese, British, and Russian populations for both Omicron variants (chi-square test, *p*-values = 0.684 and 0.206 for EG.5 and XBB.1.16, respectively) (Table 1).

**Table 1:** Comparison of mutated Spike peptide-HLA binding affinities among populations in different SARS-CoV-2 variants

Binding affinity	Omicron EG.5				Omicron XBB.1.16			
	Taiwanese	British	Russian	*p*-value^a^	Taiwanese	British	Russian	*p*-value^a^
HLA class I								
Strong, n (%)	13 (11.7%)	18 (12.7%)	16 (12.1%)	0.684	16 (11.9%)	15 (9.1%)	14 (9%)	0.206
Medium, n (%)	66 (59.5%)	72 (50.7%)	75 (56.8%)		79 (59%)	80 (48.5%)	83 (53.5%)	
Weak/no, n (%)	32 (28.8%)	52 (36.6%)	41 (31.1%)		39 (29.1%)	70 (42.4%)	58 (37.4%)	
HLA class II								
Strong, n (%)	140 (13.4%)	123 (6.9%)	211 (15.4%)	**8.102 × 10**^–14*^	118 (10.5%)	136 (6.6%)	89 (5%)	**2.4 × 10**^–08*^
Medium, n (%)	670 (63.9%)	1188 (66.8%)	883 (64.3%)		769 (68.5%)	1413 (68.2%)	1292 (72.9%)	
Weak/no, n (%)	238 (22.7%)	468 (26.3%)	280 (20.4%)		236 (21%)	524 (25.3%)	391 (22.1%)	

^a^The *p*-value was calculated using the chi-squared test and was marked with an ‘*’ if statistically significant (*p*-value < 0.05)

For HLA class II, significant differences were observed in the proportion of mutated Spike peptides with tight binding affinities to common HLA alleles from both EG.5 and XBB.1.16 across the three populations (Fig. 2B). The British population had lower proportions of tight binding affinities and relatively higher proportions of weak/no-binding affinities in mutated Spike peptides from both EG.5 and XBB.1.16 compared to the Taiwanese and Russian populations. The Russian population showed the lowest proportion of tightly binding to common HLA class II alleles for XBB.1.16, in comparison to the other two populations. The differences were supported by additional statistical analyses (chi-square test, *p*-values = 8.1 × 10^–14^ and 2.4 × 10^–08^ for EG.5 and XBB.1.16, respectively) (Table 1).

When comparing binding affinities of mutate Spike peptides to common HLA alleles between the Omicron variants across different populations, significant differences were found in the distribution of mutated Spike peptide-HLA class II binding affinity strengths between EG.5 and XBB.1.16 (chi-square test, *p*-values = 0.047, 0.656, and 1.4 × 10^–21^ for HLA class II in Taiwanese, British, and Russian populations, respectively). In contrast, the binding affinity profiles for HLA class I did not show statistical differences between the two Omicron variants (chi-square test, *p*-values = 0.997, 0.441, and 0.444 for HLA class I in Taiwanese, British, and Russian populations, respectively) (Table 2). These findings indicate that the binding affinities of mutated Spike peptides to common alleles of HLA class II are more diverse than those to HLA class I across the three populations and between the two Omicron variants.

**Table 2:** Comparison of mutated Spike peptide-HLA binding affinities between SARS-CoV-2 variants in different populations

Binding affinity	Taiwanese			British			Russian		
	EG.5	XBB.1.16	*p*-value^a^	EG.5	XBB.1.16	*p*-value^a^	EG.5	XBB.1.16	*p*-value^a^
HLA class I									
Strong, n (%)	13 (11.7%)	16 (11.9%)	0.997	18 (12.7%)	15 (9.1%)	0.441	16 (12.1%)	14 (9%)	0.444
Medium, n (%)	66 (59.5%)	79 (59%)		72 (50.7%)	80 (48.5%)		75 (56.8%)	83 (53.5%)	
Weak/no, n (%)	32 (28.8%)	39 (29.1%)		52 (36.6%)	70 (42.4%)		41 (31.1%)	58 (37.4%)	
HLA class II									
Strong, n (%)	140 (13.4%)	118 (10.5%)	0.047*	123 (6.9%)	136 (6.6%)	0.656	211 (15.4%)	89 (5%)	**1.374 × 10**^–21*^
Medium, n (%)	670 (63.9%)	769 (68.5%)		1188 (66.8%)	1413 (68.2%)		883 (64.3%)	1292 (72.9%)	
Weak/no, n (%)	238 (22.7%)	236 (21%)		468 (26.3%)	524 (25.3%)		280 (20.4%)	391 (22.1%)	

^a^The *p*-value was calculated using the chi-squared test and was marked with an ‘*’ if statistically significant (*p*-value < 0.05)

### Comparison of the binding affinity changes of mutated Spike peptides to common HLA alleles between SARS-CoV-2 Omicron variants and across different populations

**Fig. 3:**
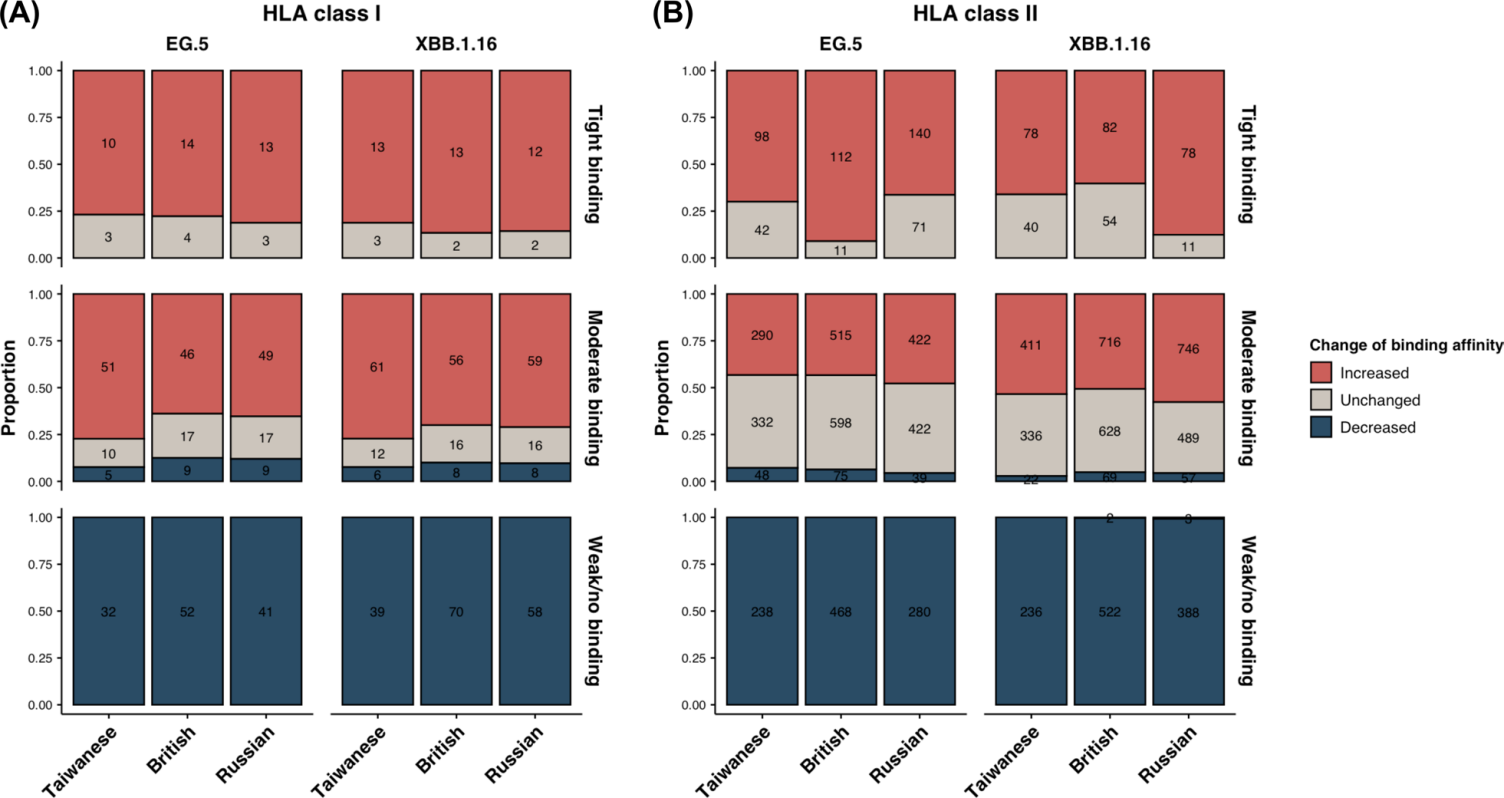
Distributions of mutated Spike peptide-common HLA binding affinity changes of SARS-CoV-2 Omicron variants across different populations. The changes in Spike peptide-common HLA class I (**A**) and II (**B**) binding affinities from the original Wuhan strain to the Omicron EG.5 and XBB.1.16 variants in Taiwanese, British, and Russian populations were determined as increased (red), decreased (blue), or unchanged (gray). Each mutated Spike peptide-common HLA binding affinity in the Omicron variants was categorized as either tight binding, moderate binding, or weak/no binding. The proportions of mutated Spike peptide-common HLA binding affinity changes of different binding affinity strengths of individual Omicron variants in different populations were illustrated in separate stacked bars

In addition to examining the mutated Spike peptide-common HLA binding affinity strengths, we also compared the changes in these affinities across Taiwanese, British, and Russian populations for EG.5 and XBB.1.16. For HLA class I, no significant differences were observed across the three populations or between the two Omicron variants in the binding affinity changes of mutated Spike peptide-common HLA (Figs. 1A–C and 3A). Most mutated Spike peptides with tight binding affinities originally had moderate or weak/no binding in the Wuhan strain, with only a small fraction retaining unchanged tight binding before the mutations occurred. This pattern was also seen in mutated Spike peptides with moderate binding affinities. Notably, only a few mutated peptides were associated with the decreased binding affinities from the original Wuhan strain. Mutated Spike peptides with weak/no binding affinities typically evolved from peptides with tight or moderate binding in the original Wuhan strain. For HLA class II, significant differences were found in the binding affinity changes of mutated Spike peptides across the three populations and between the two Omicron variants (Figs. 1D–F and 3B). Similar to HLA class I, most mutated Spike peptides with tight binding affinities to common HLA class II alleles originated from those with lower, primarily moderate, binding affinities in the original Wuhan strain. The proportion of mutated Spike peptides with unchanged tight binding affinities was comparably low for EG.5 in the British population and XBB.1.16 in the Russian population. In contrast, higher proportions of unchanged tight binding affinities to common HLA class II alleles were observed in the Taiwanese and Russian populations for EG.5 and in the Taiwanese and British populations for XBB.1.16. This led to a decrease in the proportions of tight binding affinities by increasing. For the mutated Spike peptides with moderate binding affinities to common HLA class II alleles, the distribution differed from those of HLA class I. The proportions of moderate binding mutated peptides, resulting from increased or unchanged binding affinities to population-specific common HLA class II, were comparable for both EG.5 and XBB.1.16. Mutated peptides with moderate binding affinities to common HLA class II alleles by decreasing from the Wuhan strain was rare among moderate-binding mutated peptides. However, no significant differences were found in the binding affinity changes of moderate-binding mutated peptides across the three populations or between the two Omicron variants. Mutated peptides with weak/no binding affinities to common HLA class II mainly shifted from tight and moderate in the original Wuhan strain to weak/no in the Omicron variants. Only a very small number of weak/no-binding mutated peptides retained their weak/no binding affinities to common alleles of HLA class II in the original Wuhan strain. Our findings reveal significant differences across different populations and between different SARS-CoV-2 variants in the change of binding affinities of Spike peptides after mutations to common alleles of HLA class II compared to HLA class I.

### Evaluation of the conservation and evolution of binding affinity changes of mutated Spike peptides to common HLA alleles between SARS-CoV-2 Omicron variants and across different populations

**Fig. 4:**
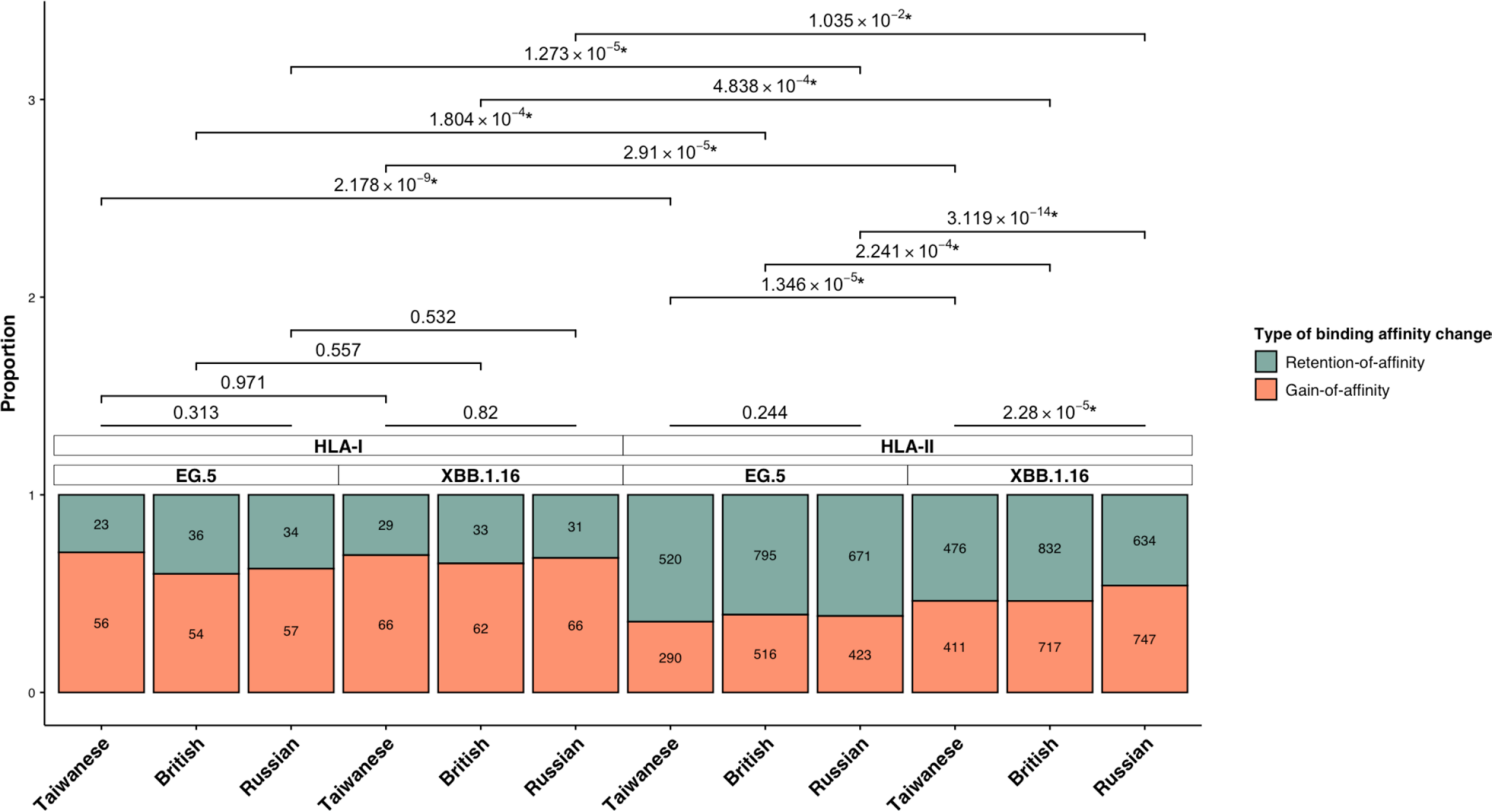
Distributions and comparisons of retention-of-affinity and gain-of-affinity changes in Spike peptide-common HLA binding affinities in SARS-CoV-2 Omicron variants across different populations. The strong and moderate binding affinities between mutated Spike peptides and common HLA class I and II alleles in Omicron EG.5 and XBB.1.16 variants were analyzed. The changes in binding affinity were classified as either retention of affinity (from tight or moderate binding in the original Wuhan strain to tight or moderate binding in the Omicron variants) or gain of affinity (from weak/no binding in the original Wuhan strain to tight or moderate binding in the Omicron variants). The proportions of retention-of-affinity and gain-of-affinity changes of individual Omicron variants in different populations were illustrated in separate stacked bars and compared using the chi-squared test. *P*-values less than 0.05 were considered significant and denoted with “*”

We next evaluated the conservation and evolution of binding affinities between mutated Spike peptides and common HLA alleles in SARS-CoV-2 variants. To this end, we focused on Spike peptide-common HLA binding affinities that changed from tight, moderate, and weak/no binding in the original Wuhan strain to tight and moderate binding in the Omicron variants. These binding affinities were classified as either retention-of-affinity type (alterations from tight or moderate binding to tight or moderate binding) or gain-of-affinity type (alterations from weak/no binding to tight or moderate binding). We compared their proportions across Taiwanese, British, and Russian populations, between the Omicron variant EG.5 and XBB.1.16, and between HLA class I and II (Fig. 4). Consistent with our above findings, no significant differences were observed across the three populations or between the two Omicron variants in terms of retention and gain of affinity to HLA class I for both EG.5 and XBB.1.16. Additionally, no significant differences were found in the distribution of such changing patterns associated with HLA class II across different populations for EG.5. However, the distribution of retention-of-affinity and gain-of-affinity numbers with HLA class II for XBB.1.16 was significantly different across the three populations. Furthermore, significant differences were observed between EG.5 and XBB.1.16 in the distribution of retention of affinity and gain of affinity to HLA class II in the Taiwanese, British, and Russian populations. Lastly, comparisons across common HLA alleles revealed significant differences between HLA class I and II in the distribution of defined binding affinity changes in each of the three populations for both EG.5 and XBB.1.16.

### Comparison of sequential binding affinity changes of mutated Spike peptides to common HLA alleles between SARS-CoV-2 Omicron variants and across different populations

**Fig. 5:**
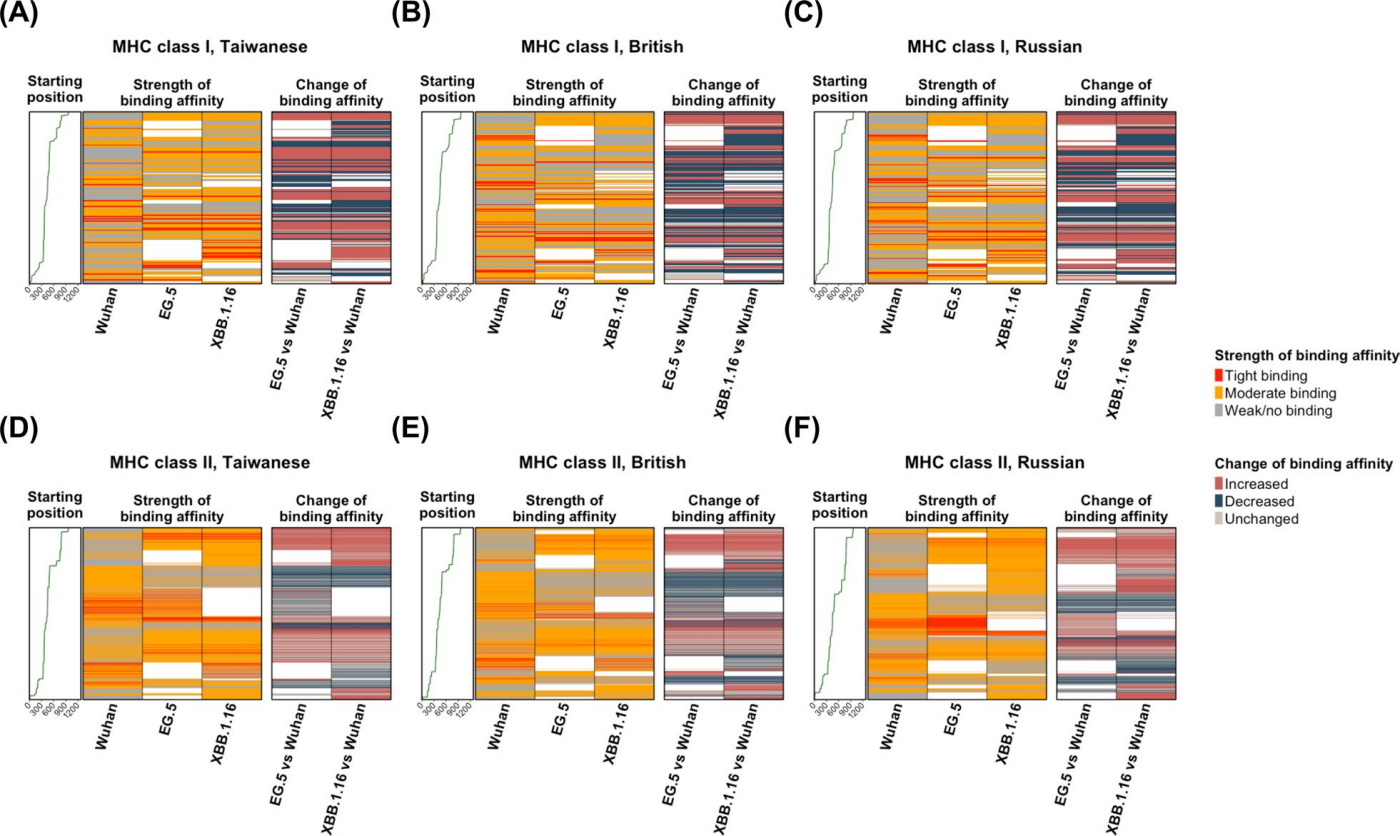
Profiles of sequential mutated Spike peptide-common HLA binding affinity changes of SARS-CoV-2 Omicron variants in different population. The strengths and changes in binding affinities of sequential Spike peptides to population-specific common HLA class I (**A**–**C**) and II (**D**–**F**) alleles in the Taiwanese (**A**, **D**), British (**B**, **E**), and Russian (**C**, **F**) populations are depicted as heatmaps. The mutated Spike peptides of the Omicron EG.5 and XBB.1.16 variants were arranged in sequential order based on their starting positions (first amino acid) of alignments to the reference Spike protein sequence of the original Wuhan strain. The strength of mutated Spike peptide-common HLA binding affinities was determined to be either tight binding (red), moderate binding (orange), or weak/no binding (gray) as displayed in the left heatmaps. The mutated Spike peptide-common HLA binding affinity change was determined as either increased (red), decreased (blue), or unchanged (gray) alteration as shown in the right heatmaps. For those Spike peptides in either Omicron variant without a mutated form, the change in binding affinity was illustrated by empty space (white) in both heatmaps

Finally, we depicted and compared the sequential binding affinities between mutated Spike peptides and common HLA alleles for SARS-CoV-2 variants across different populations. Overall, most mutated Spike peptides aligned with the central region of the Spike protein in the original Wuhan strain (Fig. 5). For HLA class I, the sequential mutated Spike peptide-common HLA binding affinity strengths and changes were similar between the British and Russian populations (Fig. 5A–C). This similarity is likely due to a higher resemblance of HLA profiles between these two populations. In contrast, the strengths and changes in binding affinity of sequential mutated Spike peptides to common HLA class II alleles varied across Taiwanese, British, and Russian populations (Fig. 5D–F). This variation is likely due to the diversity of common HLA-DPA1/DPB1 alleles among the three populations and the differences in common HLA-DRB1 profiles between the Taiwanese and British/Russian populations. Notably, sequential mutated Spike peptides in EG.5 and XBB.1.16 exhibited distinct profiles of changes in peptide-common HLA binding affinity for each HLA class in each population, likely associated with the mutational divergence of the two Omicron variants. However, many mutated Spike peptides from EG.5 and XBB.1.16 aligned to the same positions on the reference Spike protein in the original Wuhan strain were found to exhibit identical patterns of changes in peptide-common HLA binding affinities. This supports the idea that many mutations within certain Spike protein regions are shared between the two Omicron variants.

## Discussion

In this study, we conducted a comprehensive analysis of the changes in binding affinities of mutated SARS-CoV-2 Spike peptides from the EG.5 and XBB.1.16 variants with common HLA class I and class II alleles across populations from Taiwan, Britain, and Russia. Our findings underscore significant differences in how these mutations influence HLA-peptide interactions, particularly in terms of their effects on HLA class II molecules. This variance is crucial for understanding population-specific immune responses and could hold implications for the clinical management and vaccine design in response to emerging SARS-CoV-2 variants.

A key finding of our study is that mutations in the Spike protein had a greater impact on the binding affinities of HLA class II alleles compared to HLA class I alleles. This observation aligns with previous studies that emphasize the critical role of HLA class II diversity in shaping immune responses to pathogens, particularly in how effectively helper T cells recognize and respond to viral peptides [17]. HLA class II molecules, which are pivotal in presenting antigens to CD4+ helper T cells, play a central role in shaping the immune responses to SARS-CoV-2 variants [18]. The pronounced variation in class II binding affinities implies that mutations in SARS-CoV-2 may differentially affect population-specific immune responses, underscoring the role of HLA class II diversity in shaping immune protection or susceptibility.

Similar findings were reported by studies on viral epitope recognition, where HLA class I and class II molecules play crucial roles in shaping T cell responses to viruses [19, 20]. Additionally, the conservation of HLA class I binding affinities across populations, as observed in our study, suggests a conserved mechanism of cellular immunity despite viral mutations [21].

Our study also revealed both conserved and divergent changes in binding affinities between populations. While HLA class I binding affinities for mutated Spike peptides were largely consistent across Taiwanese, British, and Russian populations, there was greater variability in HLA class II binding affinities. Notably, the British population exhibited lower proportions of tightly binding mutated peptides for HLA class II alleles, particularly for the EG.5 variant. This variability in HLA class II binding affinities across populations is in line with previous research by Ren et al., who reported variability in immune responses among different COVID-19 cohorts [22]. These differences may reflect distinct population-level immune responses and emphasize the importance of HLA class II diversity in the context of evolving viral variants.

Despite the robust findings, our study has several limitations. First, it relies on computational predictions of HLA-peptide binding affinities, which, while highly informative, may not fully capture the complexities of in vivo immune responses. Additionally, our focus on common HLA alleles may overlook the contributions of rarer alleles that could significantly influence individual immune responses. Lastly, our analysis was restricted to the Spike protein, whereas other viral proteins, such as the nucleocapsid or membrane proteins, may also play critical roles in immune evasion and recognition [23]. Expanding this analysis to include a broader array of viral proteins and additional populations could provide a more comprehensive view of the global immune landscape.

In conclusion, our findings underscore the importance of HLA class II polymorphisms in shaping immune responses to emerging SARS-CoV-2 variants. The differential binding affinities observed across populations suggest that genetic diversity could significantly influence susceptibility to infection and disease outcomes. These results have important implications for vaccine development and public health strategies, particularly in designing interventions that take into account population-specific immune responses. By exploring the impact of viral mutations on HLA binding, our study contributes to the growing body of knowledge on SARS-CoV-2 evolution and its interaction with human immunity.

## Data Availability

The data supporting the study findings are available upon request from the corresponding authors.

## Abbreviations

COVID-19: Coronavirus Disease 2019
SARS-CoV-2: Severe Acute Respiratory Syndrome Coronavirus 2
HLA: Human leukocyte antigen
T-CoV: T-cell COVID-19 Atlas
